# Urinary Metal Levels, Cognitive Test Performance, and Dementia in the Multi-Ethnic Study of Atherosclerosis

**DOI:** 10.1001/jamanetworkopen.2024.48286

**Published:** 2024-12-02

**Authors:** Arce Domingo-Relloso, Katlyn E. McGraw, Susan R. Heckbert, Jose A. Luchsinger, Kathrin Schilling, Ronald A. Glabonjat, Irene Martinez-Morata, Melanie Mayer, Yongmei Liu, Alexis C. Wood, Jeff Goldsmith, Kathleen M. Hayden, Mohamad Habes, Ilya M. Nasrallah, R. Nick Bryan, Tanweer Rashid, Wendy S. Post, Jerome I. Rotter, Priya Palta, Linda Valeri, Timothy M. Hughes, Ana Navas-Acien

**Affiliations:** 1 Department of Biostatistics, Columbia University Mailman School of Public Health, New York, New York; 2 Environmental Health Sciences, Columbia University Mailman School of Public Health, New York, New York; 3 Department of Environmental Health Sciences, Columbia University Mailman School of Public Health, New York, New York; 4 Department of Epidemiology, University of Washington School of Public Health, Seattle; 5 Division of General Medicine, Columbia University Department of Medicine, New York, New York; 6 Department of Cardiology, Duke University School of Medicine, Durham, North Carolina; 7 Department of Medicine, Duke University School of Medicine, Durham, North Carolina; 8 Department of Neurology, Duke University School of Medicine, Durham, North Carolina; 9 US Department of Agriculture/Agricultural Research Service Children's Nutrition Research Center, Children’s Nutrition Research Center, Baylor College of Medicine, Houston, Texas; 10 Department of Gerontology and Geriatric Medicine, Wake Forest University School of Medicine, Winston-Salem, North Carolina; 11 Neuroimage Analytics Laboratory and the Biggs Institute Neuroimaging Core, Glenn Biggs Institute for Alzheimer’s and Neurodegenerative Diseases, University of Texas Health Science Center, San Antonio; 12 Department of Radiology, University of Pennsylvania, Philadelphia; 13 Neuroimage Analytics Laboratory, University of Texas, San Antonio, San Antonio; 14 Division of Cardiology, Department of Medicine, Johns Hopkins University School of Medicine, Baltimore, Maryland; 15 The Institute for Translational Genomics and Population Sciences, Department of Pediatrics, The Lundquist Institute for Biomedical Innovation at Harbor-UCLA Medical Center, Torrance, California; 16 Department of Radiology, University of North Carolina School of Medicine, Chapell Hill; 17 Department of Epidemiology, Harvard T.H. Chan School of Public Health, Boston, Massachusetts

## Abstract

**Importance:**

Metals are established neurotoxicants, but evidence of their association with cognitive performance at low chronic exposure levels is limited.

**Objective:**

To investigate the association of urinary metal levels, individually and as a mixture, with cognitive tests and dementia diagnosis, including effect modification by apolipoprotein ε4 allele (*APOE*4).

**Design, Setting, and Participants:**

The multicenter prospective cohort Multi-Ethnic Study of Atherosclerosis (MESA) was started from July 2000 to August 2002, with follow-up through 2018. A total of 6303 MESA participants were included. Data analysis was performed from October 12, 2023, to June 13, 2024.

**Exposure:**

Urine samples were collected at baseline (2000-2002), and arsenic, cadmium, cobalt, copper, lead, manganese, tungsten, uranium, and zinc levels were measured in 2020-2022.

**Main Outcomes and Measures:**

Digit Symbol Coding (DSC) (n = 3819) (possible score range, 0-133), Cognitive Abilities Screening Instrument (CASI) (n = 3918) (possible score range, 0-100), and Digit Span (DS) (n = 4176) (possible score range, 0-30) cognitive tests were administered in 2010-2012; higher scores of each test indicate increasing levels of positive response.

**Results:**

A total of 6303 participants were followed up for dementia diagnosis through 2018. The median age at baseline was 60 (IQR, 53-70) years, and 3303 participants (52.4%) were female. The median cognitive scores were 51 (IQR, 38-64) for DSC, 90 (IQR, 84-95) for CASI, and 15 (IQR, 12-18) for DS. There were 559 cases of dementia through the follow-up period. Inverse associations with DSC were identified: mean differences in *z* scores per IQR increase in metal levels were −0.03 (95% CI, −0.07 to 0.00) for arsenic, −0.05 (95% CI, −0.09 to −0.004) for cobalt, −0.05 (95% CI, −0.07 to −0.02) for copper, −0.04 (95% CI, −0.08 to −0.001) for uranium, and −0.03 (95% CI, −0.06 to −0.01) for zinc. Among 1058 *APOE*4 carriers, manganese was also inversely associated with DSC. The joint mean difference of DSC comparing percentile 95th with the 25th of the 9-metal mixture was −0.30 (95% CI, −0.47 to −0.14) for *APOE4* carriers and −0.10 (95% CI, −0.19 to −0.01) for noncarriers. Arsenic, cadmium, cobalt, copper, tungsten, uranium, and zinc were individually associated with dementia, with hazard ratios per IQR of metal ranging from 1.15 (95% CI, 1.03-1.29) for tungsten to 1.46 (95% CI, 1.06-2.02) for uranium. The joint hazard ratio of dementia comparing percentiles 95th with the 25th of the 9-metal mixture was 1.71 (95% CI, 1.24-3.89), with no significant difference by *APOE4* status.

**Conclusions and Relevance:**

In this study, participants with higher concentrations of metals in their urine, compared with those with lower concentrations, had worse performance on cognitive tests and greater likelihood of developing dementia. The findings of this multicenter multiethnic cohort study might inform screening and potential interventions for prevention of dementia based on individuals’ metal exposure levels and genetic profiles.

## Key Points

**Question:**

Are urinary metal levels associated with cognitive test performance and dementia, with differential associations for apolipoprotein ε4 allele (*APOE*4) carriers and noncarriers?

**Findings:**

In this cohort study including more than 6000 participants, urinary metal levels were inversely associated with speed of mental operations as measured by the Digit Symbol Coding test and positively associated with future risk of dementia. The mean difference in Digit Symbol Coding associated with metals was higher in absolute value among *APOE*4 allele carriers than among noncarriers.

**Meaning:**

These findings could inform early screening and personalized interventions for dementia prevention based on individuals’ metal exposure and genetic profiles.

## Teaser

This cohort study examines urinary metal levels, cognitive measures, and dementia diagnosis in individuals with and without apolipoprotein ε4 alleles.

## Introduction

Environmental metals are neurotoxic at high levels and induce motor and cognitive deficits at environmentally relevant doses.^1,2,3,4^ Evidence is strongest for lead, which has been associated with altered hippocampal and total brain volume and with reduced executive function and other neurologic outcomes.^5,6,7,8,9,10^ Experimental studies have also suggested that lead affects synaptic plasticity in hippocampal neurons of young rats, leading to persistent memory impairment in adulthood^11^ and induces Alzheimer disease (AD)–like pathologic changes in primates.^12^

Beyond the established neurotoxicity of lead, evidence is growing on the role of essential (copper, manganese, iron, and zinc) and nonessential (cadmium, mercury, and nickel) metals in cognitive impairment and dementia.^13,14^ Nonessential metals can alter the regulation of essential metals in the brain—a phenomenon known as metal dyshomeostasis—increasing the number of free ions in extracellular brain tissue, enhancing oxidative stress and neuroinflammation, and contributing to amyloid β misfolding and hyperphosphorylation of tau protein, the pathologic hallmarks of AD.^2,15,16,17,18^ Metals, moreover, have well-established adverse vascular effects, including endothelial dysfunction, increased blood pressure, and atherosclerosis,^19,20^ which might also contribute to dementia risk.^21^

The ε4 allele of the apolipoprotein E (*APOE4*) is a strong genetic risk factor for late onset of AD^22^ and is a common isoform of *APOE*; 25% of the global population carries at least 1 allele of that isoform.^23^ Patients with AD who carry *APOE4* show worse cognitive outcomes compared with individuals with AD who are noncarriers. Metals such as lead, copper, and zinc are cations and can bind to the *APOE4* (ApoE) isoform with lower affinity, compared with other ApoE isoforms.^24,25,26^ In a relatively small study of older adults, lead exposure was associated with lower global cognition scores among carriers of *APOE4*, but not among noncarriers.^27^ Experimental studies also support interactions between lead and *APOE4* in mice.^28^ Few studies have evaluated the association of metals and metal mixtures with cognitive impairment in general populations, including the evaluation of potential effect modification by the presence of *APOE4*.

We investigated the association of baseline metals measured in urine and both cognitive test scores and time to dementia diagnosis in participants from the Multi-Ethnic Study of Atherosclerosis (MESA). We prioritized the essential metals cobalt, copper, manganese and zinc, and the nonessential metals arsenic, cadmium, lead, tungsten and uranium. These metals were evaluated individually and as a joint metal mixture (introducing all of them in the same model). We assessed potential moderation of the association by *APOE4* genotype. We compared the effect estimates obtained for the association between metals and cognitive test scores or dementia diagnosis with those obtained for age and educational level, which are known risk factors for cognitive decline.^29,30^

## Methods

### Study Population

MESA is a multicenter, multiethnic, prospective cohort study designed to investigate the progression from subclinical to clinical cardiovascular disease (CVD).^31^ Between July 2000 and August 2002, 6814 participants were recruited at 6 study centers in Baltimore, Maryland; Chicago, Illinois; Los Angeles, California; New York, New York; St Paul, Minnesota; and Winston-Salem, North Carolina. Participants were free of diagnosed CVD, aged 45 to 84 years, and from 4 self-identified racial and ethnic groups (Black, Chinese, Hispanic, and White). For this study, we used urinary metal data collected at baseline (2000-2002) and, for a subset of participants, at examination 5 (April 2010 to February 2012), and cognitive test performance data collected at examination 5. In addition, we used time-to-event data on dementia diagnosis collected through 2018. The institutional review board at each study site approved the study and all participants gave informed consent and received financial compensation. This study follows the Strengthening the Reporting of Observational Studies in Epidemiology (STROBE) reporting guideline for cohort studies.

The inclusion criteria of this study are detailed in eFigure 1 and the eMethods in Supplement 1. After exclusion of participants with missing data, 3819 participants were included for Digit Symbol Coding (DSC), 3918 for Cognitive Abilities Screening Instrument (CASI), and 4176 for the Digit Span (DS) test. Participants included in these analyses had similar baseline characteristics to those included in the full sample (eTable 1 in Supplement 1). In addition, we compared baseline urine metals and time to dementia in 6303 participants (559 dementia cases) with data on metals, dementia, and relevant covariates.

### Urinary Metal Levels

Details of metal measurements in MESA have been described previously.^32^ Briefly, spot urine samples were collected in urine cups in the morning of the baseline examination (2000-2002) and aliquoted for transportation and long-term storage. Metals were shipped to Columbia University in 2019 and stored at −20 °C. Between 2020 and 2022, more than 15 trace elements were measured at the Trace Metals Core Laboratory at Columbia University, using inductively coupled plasma mass spectrometry with dynamic reaction cell (NexION 350S; Perkin Elmer). For this study, we prioritized metals with prior evidence of an association with cognitive function or CVD, as CVD has been associated with AD and dementia,^21^ and included arsenic, cadmium, cobalt, copper, lead, manganese, tungsten, uranium, and zinc in the analyses. However, the results for nonpriority metals are also included in eTable 4 in Supplement 1 (barium, cesium, molybdenum, strontium, and thallium). Arsenic species were measured using anion-exchange high-performance liquid chromatography followed by inductively coupled plasma mass spectrometry. We used inorganic arsenic corrected for arsenobetaine for analyses, as arsenobetaine is an organic arsenic form that has shown to be of low toxicity (hereinafter, termed arsenic).^33^ Approximately 10% of the samples were prepared and measured in duplicate to determine intraprecision, and approximately 10% were prepared and measured on different days to determine interprecision. The intraassay coefficients of variation ranged from 2.5% for zinc to 14% for uranium, and the interassay coefficients of variation ranged from 5.8% for cadmium to 16% for uranium. Samples below the instrument limits of detection (LOD) were replaced by the LOD divided by the square root of 2. The proportion of values below the LOD was less than 1% for all metals except uranium (11.7%), tungsten (32.7%), and manganese (21.3%).^32^ To correct for urine dilution, we divided metal concentrations by urine creatinine concentrations and express them as micrograms of metal per gram of creatinine. Urine creatinine level was measured with the Jaffe reaction method.^34^

### Brain Health Outcomes

Details of standardized and validated neuropsychological tests, which were conducted in the preferred language of the participants (English, Spanish, Mandarin, or Cantonese) during MESA examination 5 have been published.^35^ The DSC test is a subtest of the Wechsler Adult Intelligence Scale-III^36^ and measures the speed at which simple mental operations can be performed. This test, along with working memory, has been shown to explain a large proportion of age-related variation in memory,^37,38^ reasoning,^38^ and other cognitive abilities.^39^ The test displays a series of 9 simple symbols (eg, +,>) paired with numbers from 1 to 9. For 2 minutes, participants are asked to copy the corresponding symbol into empty boxes located below randomly ordered numbers. The final DSC score is the number of correct symbols, ranging from 0 to 133. The CASI test aims to measure global cognitive function.^40^ It includes 25 items representing 9 domains: attention, concentration, orientation, short-term memory, long-term memory, language, visual construction, verbal fluency, and abstraction/judgment. Scores on individual items are summed to provide an overall cognitive function score, ranging from 0 to 100, with higher scores indicating greater cognitive function. The DS test is another subtest of the Wechsler Adult Intelligence Scale-III^36^ and assesses working memory. In this test, the participant is asked to repeat gradually increasing spans of numbers (eg, 2-7-4), first forward and then backward. We combined backward and forward DS scores as done in previous work.^41^ The final DS score ranges from 0 to 30. We additionally evaluated a global cognitive composite score, which was calculated as a median of the *z* scores of the DSC, CASI, and DS tests.

Probable dementia, including AD, vascular dementia, and other dementias, was identified using hospitalization discharge diagnoses codes through 2018, according to the *International Classification of Diseases, Ninth Revision* (*ICD-9*): 290, 294, 331.0, 331.1, 331.2, 331.82, 331.83, 331.9, 438.0, and 780.93, and *International Statistical Classification of Diseases and Related Health Problems, Tenth Revision*: F00, F01, F03, F04, G30, G31 (excluding G31.2), I69.91, and R41.^26^ The set of *ICD* codes used to identify dementia included nonspecific codes, such as memory loss (*ICD-9*: 780.93) or persistent mental disorders due to conditions classified elsewhere (*ICD-9*: 294.8 and 294.9). The use of hospitalization discharge *ICD* codes for the diagnosis of dementia has been validated and used in prior publications from MESA.^42^

### Covariates

Age, sex, race and ethnicity, educational level, smoking status, and use of lipid-lowering and hypertension medications (yes or no) were collected by questionnaire at the baseline examination. Educational level was categorized as high school or lower, some college, or college degree or higher. Race and ethnicity was self-reported and categorized as Black, Chinese, Hispanic, and White. Smoking status was classified as never, former, and current smoker. Height and weight were measured to calculate body mass index. Systolic and diastolic blood pressure, low- and high-density lipoprotein cholesterol levels, and calibrated fasting plasma glucose levels were assessed using standard laboratory techniques.^43^ Diabetes was defined following the 2003 American Diabetes Association criteria.^44^ Kidney function can influence metal excretion in urine; therefore, we adjusted the models for estimated glomerular filtration rate (eGFR), which was calculated using the new creatinine and cystatin-C based Chronic Kidney Disease Epidemiology Collaboration equation.^45^ Genotyping procedures and DNA isolation have been described elsewhere.^46^ Genotyping was conducted in MESA participants in 2013, and from those analyses, *APOE*4 isoforms were estimated from single-nucleotide variants rs429358 and rs7412. The variable was categorized into having 0 or either 1 or 2 *APOE*4 alleles. Depression was assessed using the Center for Epidemiological Studies–Depression scale, measured at examination 5, which consists of a 20-item measure to rate how often over the past week the participants experienced symptoms associated with depression, including restless sleep, poor appetite, and feeling lonely, among others.

### Statistical Analysis

Data analysis was conducted from October 12, 2023, to June 13, 2024. We calculated descriptive statistics overall and by participant characteristics. The distribution of the metals is right-skewed, so the natural logarithmic transformation was used in all analyses. All results for the individual metals are presented comparing the 75th percentile with the 25th percentile of log-transformed metals. Spearman correlations between metals were calculated. We ran separate linear regression models to assess the association between the concentration of each metal in urine at baseline and each cognitive outcome at examination 5. Metals were included in the models as continuous log-transformed variables or using restricted quadratic splines with knots in the 10th, 50th, and 90th percentiles to assess potential nonlinear associations. Splines are widely used to assess nonlinear associations in epidemiologic studies.^29^ Models were adjusted for sociodemographic factors (age, sex, race and ethnicity, study site, and educational level), smoking status, eGFR, body mass index, continuous Center for Epidemiological Studies–Depression score, and language of administration of the cognitive tests. *P* values were adjusted for multiple comparisons accounting for the 9 metals, using the Benjamini-Hochberg approach. For each metal and each cognitive outcome, we conducted stratified analyses by *APOE4* carrier status and calculated the *P* value for interaction between the metal and *APOE4*. The association between individual metals and time to dementia was assessed using Cox proportional hazards regression models adjusted for age, sex, race and ethnicity, field center, educational level, smoking status, eGFR, and body mass index. We calculated subdistribution hazards using the Fine-Gray regression models to evaluate potential competing risks for death.^13^ The Fine-Gray regression model does not censor participants who experience a competing event, such as death, allowing them to stay in the risk set. If no competing risks are present, the subdistribution hazard should be similar to the hazard ratio (HR) calculated with a regular Cox proportional hazards regression. For comparative purposes, we evaluated the association of age, a well-established risk factor for cognitive decline, with cognitive tests and with time to dementia.

Environmental exposures might have synergistic and interactive effects in the body; thus, it is important to evaluate them using advanced statistical methods that account for their joint distribution (ie, as a mixture). We used bayesian kernel machine regression (BKMR) to evaluate the mixture of the priority metals and DSC, selected as the priority outcome based on the single-model findings. BKMR flexibly evaluates the association between a mixture of pollutants and an outcome.^47,48^ All 9 priority metals considered in our study were included in the BKMR models. Metals were log-transformed and a *z* score transformation was applied. Similar to other statistical methods for variable selection, *z* score transformation needs to be applied for any variable introduced in the BKMR kernel, as having variables quantified in different scales negatively affects the performance of the kernel for variable importance evaluation. Given that the BKMR model has not been extended to survival outcomes, we used penalized Cox proportional hazards regression models with an elastic-net penalty^49^ to estimate the joint association between the overall metal mixture and dementia risk. Confidence intervals were obtained using bootstrapping.^50^

In sensitivity analyses, we further adjusted our models for CVD risk factors (systolic blood pressure, antihypertensive medications, low-density lipoprotein cholesterol level, high-density lipoprotein cholesterol level, lipid-lowering medications, and diabetes status), as CVD history influences cognitive decline.^51^ In a subset of participants who had urinary metal levels measured at examination 5 (n = 780), we evaluated the association between mean urinary metal levels at examinations 1 and 5 (as a measure of long-term exposure) and cognitive outcomes.

All analyses were conducted using the R, version 4.3.1, statistical software (R Foundation for Statistical Computing). Cox proportional hazards regression models and Fine-Gray models were run using the coxph() function in the R package survival, and BKMR was run using the kmbayes() function of the R package BKMR. Cox elastic-net models were run combining the coxph() function in the R package survival and the cv.glmnet() function in the R package glmnet. The plots were created using the packages ggplot2, PerformanceAnalytics, ltm, and RColorBrewer. The statistical significance threshold was set to *P* < .05.

## Results

The median cognitive scores were 51 (IQR, 38-64) for DSC, 90 (IQR, 84-95) for CASI, and 15 (IQR, 12-18) for DS. The median (IQR) age at baseline was 60 (IQR, 53-70) years, 3303 (52.4%) participants were female, and 3000 (47.6 %) were male (Table 1). Among 6303 participants, 559 were diagnosed with dementia during follow-up, with a median follow-up time of 11.7 (IQR, 7.8-14.3) years in individuals with and 16.8 (IQR, 13.1-17.5) years among those without dementia. Participants who developed dementia were older, more likely to have lower educational levels, had lower eGFRs, and had higher levels of cadmium, cobalt, lead, manganese, and zinc at baseline. The proportion of *APOE4* carriers was higher among those who developed dementia than among those who did not. For DSC and CASI, the scores were lower with higher urine metal concentrations (eTable 2 in Supplement 1). For DS, the scores were similar across metal quartiles. The Spearman correlations between the metals ranged from low to moderate (eFigure 2 in Supplement 1).

**Table 1.** Baseline Characteristics of MESA Participants by *ICD* Dementia Diagnosis Status Through 2018^a^

Variable	Participants, No. (%)		
	Overall (N = 6303)	No *ICD*-coded dementia (n = 5744)	*ICD*-coded dementia (n = 559)
Follow-up time, median (IQR), y	16.7 (12.2-14.2)	16.8 (13.3-17.5)	11.7 (7.8-14.3)
Age, median (IQR), y	62 (53-70)	61 (53-69)	74 (68-78)
Sex			
Female	3302 (52.4)	3020 (52.6)	282 (50.4)
Male	3001 (47.6)	2724 (47.4)	277 (49.6)
Educational level			
≤High school	2297 (36.4)	2040 (35.5)	257 (46.0)
Some college	1465 (23.2)	1342 (23.4)	123 (22.0)
≥College	2541 (40.3)	2362 (41.1)	179 (32.0)
BMI, median (IQR)	27.5 (24.5-31.1)	27.6 (24.5-31.2)	27.2 (24.7-30.7)
eGFR, median (IQR), mL/min/1.73 m^2^	77.5 (66.7-88.4)	78.4 (67.6-89.3)	70.1 (59.8-81.2)
Smoking			
Never	3183 (50.5)	2901 (50.5)	282 (50.4)
Former	2296 (36.4)	2077 (36.2)	219 (39.2)
Current	824 (13.1)	766 (13.3)	58 (10.4)
*APOE4*			
No allele	4554 (72.3)	4197 (73.1)	357 (63.9)
1 Allele	1531 (24.3)	1358 (23.6)	173 (30.9)
2 Alleles	146 (2.3)	126 (2.2)	20 (3.6)
Missing	72 (1.1)	63 (1.1)	9 (1.6)
Metals, median (IQR), μg/g			
Inorganic arsenic	0.34 (0.21-0.55)	0.34 (0.21-0.55)	0.33 (0.21-0.53)
Cadmium	0.53 (0.36-0.8)	0.53 (0.35-0.8)	0.57 (0.39-0.86)
Cobalt	0.39 (0.28-0.57)	0.39 (0.28-0.56)	0.43 (0.3-0.62)
Copper	12.5 (10.1-15.9)	12.4 (9.9-15.8)	13.6 (10.7-16.9)
Lead	0.93 (0.66-1.33)	0.93 (0.65-1.33)	0.96 (0.7-1.33)
Manganese	0.26 (0.16-0.44)	0.25 (0.16-0.43)	0.29 (0.17-0.51)
Tungsten	0.06 (0.04-0.11)	0.06 (0.04-0.11)	0.06 (0.04-0.11)
Uranium	0.005 (0.003-0.011)	0.005 (0.003-0.011)	0.005 (0.003-0.011)
Zinc	542.8 (363.5-812.6)	532.1 (358.9-796.5)	639.9 (438.1-977.6)
Test scores, median (IQR)^b^			
Digit Symbol Coding	51 (38-63)	52 (39-64)	35 (25-45)
Cognitive Abilities Screening Instrument	89.5 (84.0-94.5)	90.0 (84.4-94.8)	84.0 (77.9-89.5)
Digit Span	15 (12-18)	15 (12-18)	13 (11-16)
Global cognitive composite score	21.9 (17.2-26.5)	22.2 (17.6-26.7)	16.2 (11.9-20.6)

Abbreviations: BMI, body mass index (calculated as weight in kilograms divided by height in meters squared); eGFR, estimated glomerular filtration rate; *ICD*, *International Classification of Diseases*; MESA, Multi-Ethnic Study of Atherosclerosis.

^a^ Dementia was based on hospitalization or death with *ICD* codes for dementia.^42^ Both *International Classification of Diseases, Ninth Revision* and *International Statistical Classification of Diseases and Related Health Problems, Tenth Revision*, codes were used.

^b^ The Digit Symbol Coding score range is 0 to 133, with higher scores indicating correct identification of a larger number of symbols. The Cognitive Abilities Screening Instrument score range is 0 to 100, with higher scores indicating greater global cognitive function. The Digit Span test score range is 0 to 30, with higher scores indicating greater working memory. The global cognitive composite score is calculated as a mean of the *z* scores of these tests.

In adjusted models, baseline arsenic, cobalt, copper, uranium, and zinc levels were inversely associated with DSC levels (eTable 3 in Supplement 1); the mean differences in DSC *z* score per interquartile range (IQR) of log-transformed metal levels were −0.03 (95% CI, −0.07 to 0.00) for arsenic, −0.05 (95% CI, −0.09 to −0.004) for cobalt, −0.05 (95% CI, −0.07 to −0.02) for copper, −0.04 (95% CI, −0.08 to −0.001) for uranium, and −0.03 (95% CI, −0.06 to −0.01) for zinc. The dose-response plots suggest a potential nonlinear association with a threshold for arsenic, cobalt, copper, lead, uranium, and zinc (eFigure 3 in Supplement 1). In the subset of participants (n = 1058) carrying either 1 or 2 *APOE*4 alleles, the mean differences were lower for cobalt (mean difference, −0.084 vs −0.041), uranium (mean difference, −0.073 vs −0.023), and zinc (mean difference, −0.078 vs −0.019) compared with the mean differences among noncarriers (n = 2761). Manganese also showed an inverse association (Table 2). We also found statistically significant interactions between *APOE4* and both manganese and zinc with DSC. Arsenic was associated with DSC only among the non-*APOE*4 carriers.

**Table 2.** Digit Symbol Coding Measured in 2010-2012 Per 1-IQR Increase in Log-Transformed Urine Metal Levels^a^

Metal	*z* Score difference, mean (95% CI)^b^		*P* value for interaction^c^
	No *APOE-ε4* allele (n = 2761)	1-2 *APOE-ε4* alleles (n = 1058)^d^	
Arsenic	−0.041 (−0.081 to −0.001)^e^	−0.016 (−0.084 to 0.053)	.47
Cadmium	−0.008 (−0.055 to 0.039)	−0.049 (−0.118 to 0.019)	.12
Cobalt	−0.041 (−0.078 to −0.005)^e^^,^^f^	−0.084 (−0.164 to −0.003)^e^	.99
Copper	−0.055 (−0.087 to −0.023)^e^	−0.050 (−0.100 to 0.000)^e^^,^^f^	.59
Lead	0.019 (−0.019 to 0.056)	−0.052 (−0.110 to 0.007)	.08
Manganese	0.010 (−0.025 to 0.045)	−0.066 (−0.124 to −0.009)^e^	.02
Tungsten	−0.018 (−0.052 to 0.017)	−0.023 (−0.083 to 0.037)	.64
Uranium	−0.023 (−0.068 to 0.022)	−0.073 (−0.14 to −0.006)^e^	.09
Zinc	−0.019 (−0.052 to 0.015)	−0.078 (−0.134 to −0.023)^e^	.03

Abbreviation: *APOE*, apolipoprotein E.

^a^ Mean difference per 1-IQR increase in log-transformed urine metal levels (micrograms per gram of creatinine) measured in 2000-2002 stratified by *APOE*-ε4 carrier status. Model adjusted for age, sex, study site, educational level, estimated glomerular filtration rate, smoking status, body mass index, language of test administration, and depression score.

^b^ The SD of Digit Symbol Coding was 18.6.

^c^ *P* value for interaction between carrying 1 or 2 *APOE4* alleles and each metal.

^d^ Participants carrying either 1 or 2 *APOE4* alleles.

^e^ Metals associated with the Digit Symbol Coding outcome.

^f^ Effect estimates and *P* values obtained from a model using restricted quadratic splines with knots in the 10th, 50th, and 90th percentiles of the metal distribution.

Cobalt, copper, and zinc were associated with the global cognitive score. Urinary lead showed a positive association with CASI, and copper showed an inverse association with DS. None of the other metals were associated with CASI or DS (eTable 3 in Supplement 1). eTable 4 in Supplement 1 reports the association between nonpriority metals and cognitive tests. Cesium was positively associated with CASI and the global cognitive score, and thallium also showed a positive association with CASI. None of the nonpriority metals showed associations with decreased cognitive function.

**Figure 1.**
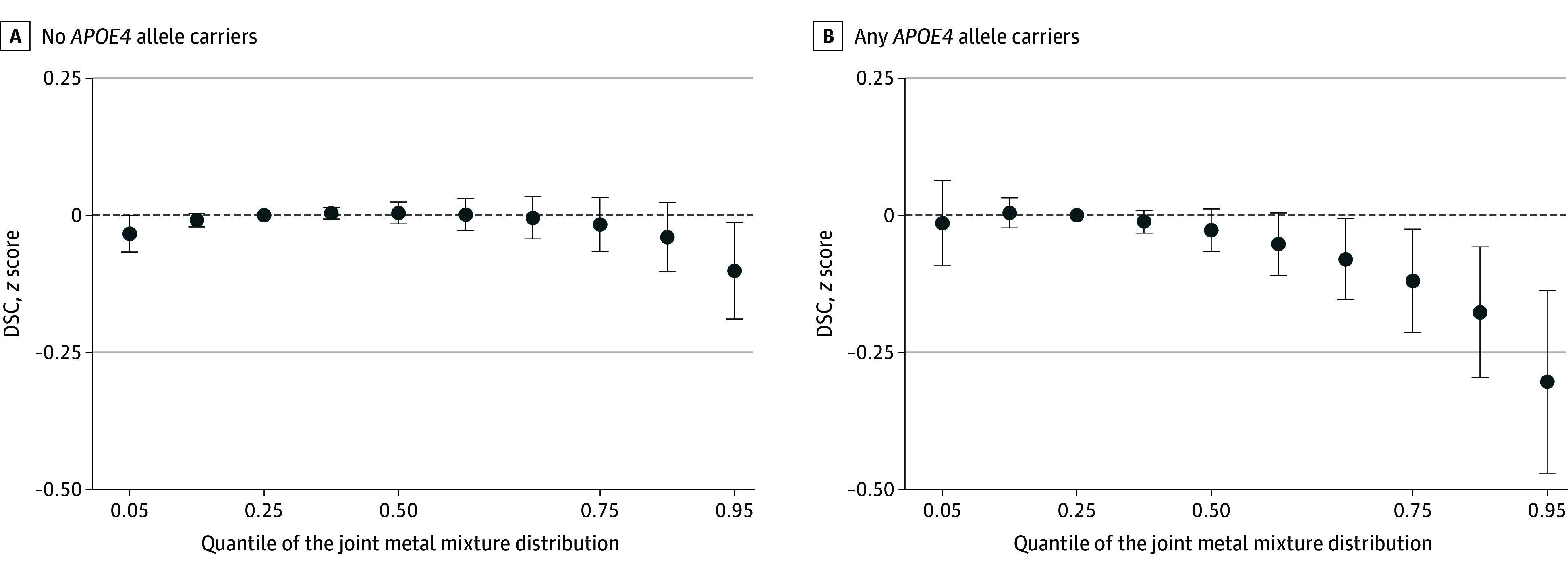
Mean Difference of *z* Score Digit Symbol Coding for the Overall Urine Metal Mixture Distribution Distribution of 9 metals (arsenic, cadmium, cobalt, copper, lead, manganese, tungsten, uranium, and zinc) using bayesian kernel machine regression for no *APOE4* allele carriers (A) and any *APOE4* allele carriers (B). Mean difference in *z*-score of the Digit Symbol Coding test when fixing all metal levels to their 25th percentile, compared with fixing them to different percentiles displayed on the x-axis. Covariates are fixed to their median (for continuous variables) or mode (for categorical variables). Error bars indicate 95% credibility intervals.

The metal mixtures analysis focused on the DSC test since there were no associations with CASI and DS for more than 1 metal. Figure 1 shows the association of the overall metal mixture with DSC when all metals were fixed to different percentiles (ranging from the 5th to 95th percentile), compared with when all metals were fixed to the 25th percentile. We found a marked and inverse association between the 9-metal mixture and DSC in the subset of participants carrying either 1 or 2 *APOE4* alleles. The findings in the subset of participants with no *APOE4* alleles were largely not statistically significant, except at the highest levels of the mixture. The mean difference (95% credibility intervals) of DSC in *z* score comparing the whole mixture in the 95th percentile to the 25th percentile was −0.30 (95% credibility interval, −0.47 to −0.14) for *APOE4* carriers and −0.10 (95% credibility interval, −0.19 to −0.01) for non-*APOE4* carriers. eFigure 4 in Supplement 1 shows the individual dose-response association of each metal in the presence of the other metals, as well as the posterior inclusion probabilities, which indicate the variable importance of each metal in the model.

In sensitivity analyses, arsenic, cobalt, copper, and uranium were associated with DSC after adjustment for CVD risk factors (eTable 5 in Supplement 1). For zinc, the comparison with DSC was not statistically significant after adjustment for CVD risk factors; however, the interaction between zinc and *APOE4* with DSC was statistically significant. For the subset of participants who had urinary metal levels measured at examination 5, the results were mostly consistent (eTable 6 in Supplement 1).

**Figure 2.**
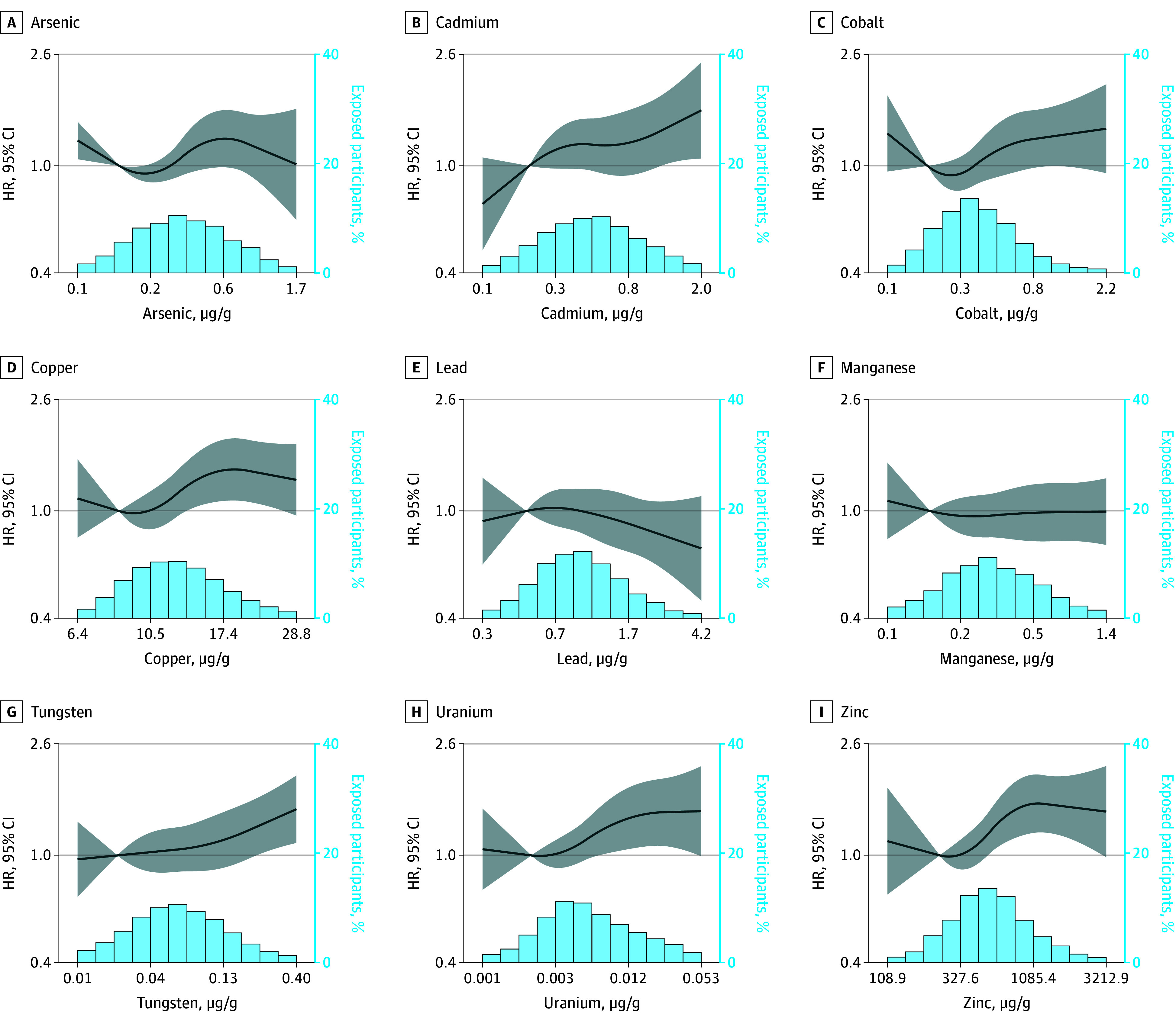
Dose-Response of the Association Between Urinary Metal Levels and Time to Dementia Using Restricted Quadratic Splines The values of the x-axis are in logarithmic scale, but the axis labels are not log-transformed and represent the real concentrations of urinary metal levels. The y-axis is in *z* score scale. The solid lines represent adjusted hazard ratios (HRs) based on restricted quadratic splines for the log-transformed concentration of urinary metal levels, with knots set at the 10th, 50th, and 90th percentiles. The shadowed areas represent the upper and lower 95% CIs. The bars represent histograms of the distribution of each urinary metal. The reference was set at the 10th percentile. The x-axis represents metal concentrations at their original scale, and the z-axis represents the percentage of participants exposed to each range of metal concentrations in the displayed histogram. Models were adjusted for age, sex, race and ethnicity, study site, educational level, estimated glomerular filtration rate, smoking status, and body mass index.

Associations with dementia risk (Table 3) were noted with HRs per IQR increase of log-transformed metal levels: HR, 1.32 (95% CI, 1.07-1.65) for arsenic; HR, 1.17 (95% CI, 1.01-1.36) for cadmium; HR, 1.18 (95% CI, 1.01-1.38) for cobalt; HR, 1.16 (95% CI, 1.05-1.28) for copper; HR, 1.15 (95% CI, 1.03-1.29) for tungsten; HR, 1.46 (95% CI, 1.06-2.02) for uranium; and HR, 1.35 (95% CI, 1.13-1.61) for zinc. The dose-response plots suggest nonlinear associations (Figure 2). There was no evidence of moderation of the association between individual metals and dementia by *APOE4*. The HR of dementia comparing the 95th with the 25th percentiles of the whole metal mixture was 1.71 (95% CI, 1.24-3.89). Among *APOE4* carriers, the HR for the whole mixture was 1.70 (95% CI, 0.67-3.92), whereas for the non-*APOE4* carriers, the HR was 3.03 (95% CI, 1.39-6.22). The subdistribution hazards from the Fine-Gray model were slightly attenuated but similar, which did not provide evidence of competing risks (eTable 7 in Supplement 1).

**Table 3.** Possible *ICD*-Based All-Cause Dementia During 14.2 Years of Mean Follow-Up Per 1-IQR Increase in Log-Transformed Urine Metal Levels^a^^,^^b^

Metal	Hazard ratio (95% CI)			*P* value for interaction^c^
	Overall (559 cases/5744 noncases)	No *APOE*-ε4 allele (357 cases/4197 noncases)	1-2 *APOE*-ε4 alleles (193 cases/1484 noncases)^d^	
Arsenic	1.32 (1.07 to 1.65)^e^^,^^f^	0.96 (0.82 to 1.13)	1.15 (0.92 to 1.45)	.16
Cadmium	1.17 (1.01 to 1.36)^e^	1.39 (1.15 to 1.68)^e^	0.87 (0.67 to 1.13)	.78
Cobalt	1.18 (1.01 to 1.38)^e^^,^^f^	1.13 (0.99 to 1.30)	1.05 (0.87 to 1.27)	.44
Copper	1.16 (1.05 to 1.28)^e^^,^^f^	1.20 (1.06 to 1.37)^e^^,^^f^	0.99 (0.83 to 1.19)	.85
Lead	0.92 (0.82 to 1.04)	0.90 (0.78 to 1.04)	0.93 (0.77 to 1.14)	.25
Manganese	1.00 (0.90 to 1.12)	0.99 (0.87 to 1.14)	1.00 (0.82 to 1.23)	.30
Tungsten	1.15 (1.03 to 1.29)^e^	1.09 (0.95 to 1.26)	1.24 (1.03 to 1.49)^e^	.15
Uranium	1.46 (1.06 to 2.02)^e^^,^^f^	1.74 (1.14 to 2.65)^e^^,^^f^	0.98 (0.78 to 1.23)	.25
Zinc	1.35 (1.13 to 1.61)^e^^,^^f^	1.34 (1.07 to 1.67)^e^^,^^f^	1.24 (1.03 to 1.49)^e^	.70

Abbreviations: *APOE*, apolipoprotein E; *ICD*, *International Classification of Diseases.*

^a^ Dementia diagnosis per 1-IQR increase in log-transformed urine metal levels (micrograms per gram of creatinine) measured in 2000-2002.

^b^ Model adjusted for age, sex, race and ethnicity, study site, educational level, estimated glomerular filtration rate, smoking status, and body mass index. Both *International Classification of Diseases, 9th Revision* and *International Statistical Classification of Diseases and Related Health Problems, 10th Revision*, codes were used.

^c^ *P* value for interaction between carrying 1 or 2 *APOE*4 alleles and each metal.

^d^ Participants carrying either 1 or 2 *APOE4* alleles.

^e^ Metals associated with dementia.

^f^ Effect estimates and *P* values obtained from a model using restricted quadratic splines with knots in the 10th, 50th, and 90th percentiles of the metal distribution.

The mean difference DSC in *z* score for a 1-year increase in age was −0.047 (95% CI, −0.050 to −0.044). For dementia, the HR for a 1-year increase in age was 1.16 (95% CI, 1.15-1.18).

## Discussion

In this multiethnic study, we found that higher levels of metals, individually and as a mixture, were associated with lower cognitive performance as evaluated by the DSC test, with a lower mean difference among *APOE4* carriers than among noncarriers. The association of DSC, a measure of speed of mental operations, with an increase in the 9-metal mixture from the 25th to the 95th percentiles, was comparable with the association of a 6-year increase in age with the outcome, which is an important, but not modifiable, risk factor for cognitive decline. For non-*APOE4* carriers, the finding with DSC was only significant in the high range of the 9-metal mixture. We also found a prospective association between baseline urinary metal levels, individually and as a mixture, and future risk for dementia, with no evidence of moderation by *APOE4* carrier status in the hypothesized direction. The risk observed when comparing the 75th vs 25th levels of arsenic, uranium, and zinc with dementia was comparable with the risk associated with a 2-year increase in age, whereas the association with cadmium, cobalt, copper, and tungsten was comparable with a 1-year increase in age.

Carrying the *APOE4* allele increases the risk of AD, which highlights the need to identify prevention strategies targeting modifiable risk factors among *APOE4* carriers.^22^ Potential interactions between individual metals, such as zinc, copper, lead, and cadmium, with the ApoE4 protein in cognitive decline and AD have been reported, although prior studies have been small and focused on single metals.^3,4,5,6^ A recent study summarized biological evidence showing potential interactions between metals and the ApoE4 protein.^52^ Metals might accumulate in amyloid plaques, leading to metal dyshomeostasis, which can result in decreased ApoE levels in AD. Decreased ApoE levels in *APOE4* carriers are considered an important factor for AD onset and development.^53^ In addition, metals have a lower ability to stabilize ApoE4 isoforms compared with ApoE2 and ApoE3, which might make ApoE4 isoforms more susceptible to proteolytic degradation and subsequently to disruption of mitochondrial and cytoskeletal processes, resulting in neurodegeneration.^54^ We found smaller mean differences with DSC for cobalt, manganese, uranium, and zinc for *APOE4* carriers, despite the larger sample size in the non-*APOE4* group, and statistically significant interactions for *APOE4* with manganese and zinc. Arsenic was the only metal for which the mean difference was smaller among non*-APOE4* carriers, which could be related to the fact that arsenic is present as an oxoanion, not as a cation, and the differential binding of ApoE isoforms with metals has been found for cations.^24,25,26^ Our results point out that increased urinary metal levels might interact with *APOE4* to increase the risk of developing cognitive decline, which is in line with the biological hypothesis of previous studies.^55^ Future experimental studies would be useful to evaluate this gene-environment interaction, as exposure to metals is a modifiable risk factor.

We found associations with DSC and dementia for both essential and nonessential metals in urine. Increases in urinary excretion of essential metals might not reflect excess intake of those elements, but loss of body reserves due to disruption of other biological processes.^56^ Several studies support that both low and high levels of copper are associated with cognitive decline and AD.^57,58^ In addition, there is evidence for a role of copper dyshomeostasis not only in AD,^59^ but also in type 2 diabetes, which might influence cognitive deterioration.^60^ Less is known about cobalt, although some evidence of neurotoxicity exists in animals^61^ and humans.^62^ Urinary cobalt was also associated with cognitive impairment in a study of 840 participants from the National Health and Nutrition Examination Survey in 2011-2014.^63^ Our study supports and extends this observation by reporting associations between urinary cobalt levels and both cognitive test scores and dementia. For zinc, both deficiency and excess in the brain have been associated with cognitive decline,^64^ and urinary zinc losses are also associated with diabetes and prediabetes.^65^

Regarding nonessential metals, urinary arsenic, cadmium, and tungsten were associated with poor performance on some cognitive tests in US adults in the National Health and Nutrition Examination Survey 2011-2014.^63^ Arsenic is associated with brain inflammatory responses^66^ and has been linked to reduced memory and intellectual abilities,^67^ even at low levels.^68^ Previous studies have reported that arsenic exposure increases β-amyloid protein and induces hyperphosphorylation of tau protein, oxidative stress, inflammation, endothelial cell dysfunction, and angiogenesis.^67^ In addition, arsenic might induce hippocampal morphologic changes.^67^ Cadmium has been associated with cognitive impairment and AD in several studies.^69,70,71^ Potential physiologic mechanisms for cadmium neurotoxicity might involve cadmium-induced neuronal cell apoptosis, increasing permeability of the blood-brain barrier, and oxidative stress.^72^ However, both arsenic and cadmium are associated with cardiovascular disease,^73^ which is an important risk factor for vascular dementia.^21^ Less is known about the potential brain health effects of uranium and tungsten. In this study, we found smaller mean differences among *APOE4* carriers than noncarriers for uranium, and both tungsten and uranium were associated with dementia. We used the mean concentration (micrograms per gram of creatinine) of urinary metal levels measured at examinations 1 and 5 as an estimate of long-term exposure to those metals in the 8 to 11 years immediately before cognitive testing. The associations between this measure and DSC in a smaller subset were consistent with those found for metals at examination 1, except for lead. The positive association for lead with CASI and the global cognitive score is counterintuitive given the strong evidence of lead as a neurotoxicant.^74^ This might be related to urinary lead levels being a less-established biomarker of internal dose compared with blood lead levels,^75^ which supports the need of future studies measuring lead in blood in MESA and other cohorts.

The nonlinear associations identified for most of the metals with DSC suggest evidence of a threshold effect in which metals might not be associated with cognitive performance at low levels but start to show a negative impact after a certain threshold of exposure. This nonlinear association with DSC was also apparent in the mixture models. These findings are relevant for public policy, as the exposure levels of this population are comparable to those of general populations in urban and suburban areas of the US.^76^

### Strengths and Limitations

Strengths of this study include the relatively large sample size and long follow-up, the rich covariate information, the inclusion of multiple racial and ethnic groups from different urban and suburban areas of the US, and the availability of cognitive performance tests and clinical dementia data. We also analyzed repeated urinary metal measures for a subset of the participants, which adds robustness to the exposure assessment. Our statistical methods allowed us to flexibly evaluate the metals as a mixture.

This study has limitations. In the dementia ascertainment, data on patients with dementia who were never hospitalized, died, or were lost to follow-up without a diagnosis were likely not captured. Both false-negative and false-positive findings in dementia events are likely present. Also, the dementia diagnosis currently available in MESA includes nonspecific *ICD* codes, such as memory loss, which might lead to false-positive reports. However, a previous evaluation of the validity of the *ICD*-based dementia diagnosis in MESA concluded that the validity of the dementia diagnosis was reasonable (73%-92% concordant with medical record text), which was considered the true-positive report.^77^ Future studies should evaluate specific dementia types separately, as different biological pathways might be involved. In addition, competing risks for death are an important concern in aging research, given that many participants who may otherwise develop dementia might die from other causes before a dementia diagnosis. However, the currently available statistical methods generally do not handle competing risks in an appropriate way.^78^ Future research is needed to develop statistical models to better quantify the potential biases derived from competing risks in aging research. In addition, although this was a relatively large study of the cognitive outcomes associated with metals exposure, the sample size was insufficient to evaluate the associations between metals and cognitive test scores for carriers of 2 *APOE4* alleles.

## Conclusions

In this cohort study, we found an inverse association of essential and nonessential metals in urine, both individually and as a mixture, with the speed of mental operations, as well as a positive association of urinary metal levels with dementia risk. Our results suggest inverse and differential associations between metals and DSC test scores among *APOE4* carriers and noncarriers. As metal exposure and levels in the body are modifiable, these findings could inform early screening and precision interventions for dementia prevention based on individuals’ metal exposure and genetic profiles.

## Notes

Supplement 1. **eMethods.** Detailed Methods **eTable 1.** Participant Characteristics of Included Participants Compared to the Full Sample **eTable 2.** Median (25th, 75th percentiles) of Cognitive Tests Measured in MESA Participants in 2010-2012 by Quartiles of Urine Metals per g Creatinine Measured in 2000-2002 **eTable 3.** Mean Difference (95% CI) of *z*-Score Cognitive Tests Measured in 2010-2012 per One Interquartile Range Increase in Log-Transformed Urine Metal Levels (μg/g creatinine) measured in 2000-2002 **eTable 4.** Mean Difference (95% CI) of *z*-Score Cognitive Tests Measured in 2010-2012 per One Interquartile Range Increase in Log-Transformed Urine Non-Priority Metal Levels Measured in 2000-2002 (Exam 1) **eTable 5.** Mean Difference (95% CI) of *z*-Score Digit Symbol Coding Measured in 2010-2012 per One Interquartile Range Increase in Log-Transformed Urine Metal Levels (μg/g Creatinine) Measured in 2000-2002 in MESA Participants, Overall and by *APOE4* Carrier Status, Adjusted for Cardiovascular Disease Risk Factors **eTable 6.** Mean Difference (95% CI) of *z*-Score Digit Symbol Coding Measured in 2010-2012 (Exam 5) per One Interquartile Range Increase in the Mean Between Log-Transformed Urine Metal Levels Measured in 2000-2002 (Exam 1) and 2010-2012 (Exam 5), as a Measure of Long-Term Exposure to Metals **eTable 7.** Subdistribution Hazards (95 %CIs) of Possible *ICD*-Based All Cause Dementia per One Interquartile Range Increase in Log-Transformed Urine Metal Levels Calculated From the Fine-Gray Model to Evaluate Competing Risks for Death **eFigure 1.** Flowchart of the Multi-Ethnic Study of Atherosclerosis Participants **eFigure 2.** Spearman Correlations Between Metals **eFigure 3.** Dose-Response of the Association Between Urinary Metals and Digit Symbol Coding Using Restricted Quadratic Splines **eFigure 4.** Mean Difference (95% CI) of *z*-Score Digit Symbol Coding (DSC) for the Individual Urine Metal Distribution Using Bayesian Kernel Machine Regression for Any *APOE4* Allele Carriers, No *APOE4* Allele Carriers

Supplement 2.
Data Sharing Statement
