# Errors in Funding and Affiliations

**DOI:** 10.1001/jamanetworkopen.2024.60906

**Published:** 2025-01-21

In the Original Investigation titled “Urinary Metal Levels, Cognitive Test Performance, and Dementia in the Multi-Ethnic Study of Atherosclerosis,”^1^ published December 2, 2024, National Institutes of Health grant 5R01AG07751802 should have been included as study funding for Drs Domingo-Relloso and Valeri. Dr Valeri's affiliations should have included the Department of Epidemiology, Harvard T.H. Chan School of Public Health, Boston, Massachusetts, and Dr Mayer's affiliation should have been given as the Department of Biostatistics, Columbia University Mailman School of Public Health, New York, New York. This article has been corrected.^1^
