# Supplementary material for: Urinary Metal Levels, Cognitive Test Performance, and Dementia in the Multi-Ethnic Study of Atherosclerosis
Source: JAMA Netw Open. 2024 Dec 2;7(12):e2448286. doi: 10.1001/jamanetworkopen.2024.48286 (PMC11612832; doi:10.1001/jamanetworkopen.2024.48286)
Supplement: Supplement 1. — eMethods. Detailed Methods eTable 1. Participant Characteristics of Included Participants Compared to the Full Sample eTable 2. Median (25th, 75th percentiles) of Cognitive Tests Measured in MESA Participants in 2010-2012 by Quartiles of Urine Metals per g Creatinine Measured in 2000-2002 eTable 3. Mean Difference (95% CI) of z-Score Cognitive Tests Measured in 2010-2012 per One Interquartile Range Increase in Log-Transformed Urine Metal Levels (μg/g creatinine) measured in 2000-2002 eTable 4. Mean Difference (95% CI) of z-Score Cognitive Tests Measured in 2010-2012 per One Interquartile Range Increase in Log-Transformed Urine Non-Priority Metal Levels Measured in 2000-2002 (Exam 1) eTable 5. Mean Difference (95% CI) of z-Score Digit Symbol Coding Measured in 2010-2012 per One Interquartile Range Increase in Log-Transformed Urine Metal Levels (μg/g Creatinine) Measured in 2000-2002 in MESA Participants, Overall and by APOE4 Carrier Status, Adjusted for Cardiovascular Disease Risk Factors eTable 6. Mean Difference (95% CI) of z-Score Digit Symbol Coding Measured in 2010-2012 (Exam 5) per One Interquartile Range Increase in the Mean Between Log-Transformed Urine Metal Levels Measured in 2000-2002 (Exam 1) and 2010-2012 (Exam 5), as a Measure of Long-Term Exposure to Metals eTable 7. Subdistribution Hazards (95 %CIs) of Possible ICD-Based All Cause Dementia per One Interquartile Range Increase in Log-Transformed Urine Metal Levels Calculated From the Fine-Gray Model to Evaluate Competing Risks for Death eFigure 1. Flowchart of the Multi-Ethnic Study of Atherosclerosis Participants eFigure 2. Spearman Correlations Between Metals eFigure 3. Dose-Response of the Association Between Urinary Metals and Digit Symbol Coding Using Restricted Quadratic Splines eFigure 4. Mean Difference (95% CI) of z-Score Digit Symbol Coding (DSC) for the Individual Urine Metal Distribution Using Bayesian Kernel Machine Regression for Any APOE4 Allele Carriers, No APOE4 Allele Carriers [file jamanetwopen-e2448286-s001.pdf]

Supplementary Online Content

Domingo-Relloso A, McGraw KE, Heckbert SR, et al. Urinary metals, cognitive test performance, and dementia in the Multi-Ethnic Study of Atherosclerosis. *JAMA Netw Open*. 2024;7(12):e2448286. doi:10.1001/jamanetworkopen.2024.48286

eMethods. Detailed Methods

eTable 1. Participant Characteristics of Included Participants Compared to the Full Sample

eTable 2. Median (25th, 75th percentiles) of Cognitive Tests Measured in MESA Participants in 2010-2012 by Quartiles of Urine Metals per g Creatinine Measured in 2000-2002

eTable 3. Mean Difference (95% CI) of *z*-Score Cognitive Tests Measured in 2010-2012 per One Interquartile Range Increase in Log-Transformed Urine Metal Levels (μg/g creatinine) measured in 2000-2002

eTable 4. Mean Difference (95% CI) of *z*-Score Cognitive Tests Measured in 2010-2012 per One Interquartile Range Increase in Log-Transformed Urine Non-Priority Metal Levels Measured in 2000-2002 (Exam 1)

eTable 5. Mean Difference (95% CI) of *z*-Score Digit Symbol Coding Measured in 2010-2012 per One Interquartile Range Increase in Log-Transformed Urine Metal Levels (μg/g Creatinine) Measured in 2000-2002 in MESA Participants, Overall and by *APOE4* Carrier Status, Adjusted for Cardiovascular Disease Risk Factors

eTable 6. Mean Difference (95% CI) of *z*-Score Digit Symbol Coding Measured in 2010-2012 (Exam 5) per One Interquartile Range Increase in the Mean Between Log-Transformed Urine Metal Levels Measured in 2000-2002 (Exam 1) and 2010-2012 (Exam 5), as a Measure of Long-Term Exposure to Metals

eTable 7. Subdistribution Hazards (95 %CIs) of Possible *ICD*-Based All Cause Dementia per One Interquartile Range Increase in Log-Transformed Urine Metal Levels Calculated From the Fine-Gray Model to Evaluate Competing Risks for Death

eFigure 1. Flowchart of the Multi-Ethnic Study of Atherosclerosis Participants

eFigure 2. Spearman Correlations Between Metals

eFigure 3. Dose-Response of the Association Between Urinary Metals and Digit Symbol Coding Using Restricted Quadratic Splines

eFigure 4. Mean Difference (95% CI) of *z*-Score Digit Symbol Coding (DSC) for the Individual Urine Metal Distribution Using Bayesian Kernel Machine Regression for Any *APOE4* Allele Carriers, No *APOE4* Allele Carriers

This supplementary material has been provided by the authors to give readers additional information about their work.

© 2024 Domingo-Relloso A et al. *JAMA Network Open.*

eMethods. Detailed Methods

Inclusion criteria

A total of 6,729 MESA participants had urinary metals and serum creatinine measured at baseline (Figure S1). After excluding 15 participants missing estimated glomerular filtration rate (eGFR), 21 missing smoking status, one participant missing education and two participants with extreme values for urinary copper and cobalt (100 times higher than the other highest values in the study), 6,690 participants were retained. Of those, 6,343 had data on *APOE4* genotype, and 4,651 completed MESA exam 5. We used cognitive assessments conducted at exam 5, and excluded missing values and invalid scores for each, in addition to 241 participants missing data on the Center for Epidemiological Studies-Depression (CES-D) scale, leaving 3,819 participants for Digit Symbol Coding (DSC), 3,918 participants for Cognitive Abilities Screening Instrument (CASI), and 4,176 participants for Digit Span (DS) test. We conducted a separate analysis evaluating the association between urine metals and cognitive test scores in N=780 participants with urine metals measured at exam 5 considering the mean urinary metal levels of exams 1 and 5 as a measure of long-term metal exposure. In addition, we evaluated the association between baseline urine metals and time-to-dementia in N=6,231 participants (550 dementia cases) with data on metals, dementia, and relevant covariates (Figure S1).

Brain health outcomes

The Digit Symbol Coding (DSC) test is a subtest of the Wechsler Adult Intelligence Scale-III (42) and measures the speed at which simple mental operations can be performed. This test, along with working memory, has shown to explain a large proportion of age-related variation in memory (43,44), reasoning (44), and other cognitive abilities (45). The test displays a series of nine simple symbols (e.g., +, >) paired with numbers from 1 to 9. For two minutes, participants are asked to copy the corresponding symbol into empty boxes located below randomly ordered numbers. The final DSC score is the number of correct symbols, which ranges from 0 to 133. The Cognitive Abilities Screening Instrument (CASI) test aims to measure global cognitive function (46). It includes 25 items representing nine domains: attention,

© 2024 Domingo-Relloso A et al. *JAMA Network Open.*

concentration, orientation, short-term memory, long-term memory, language, visual construction, verbal fluency, and abstraction/judgment. Scores on individual items are summed to provide an overall cognitive function score ranging from 0 to 100. The Digit Span (DS) test is another sub-test of the Wechsler Adult Intelligence Scale-III (42) and assesses working memory. In this test, the participant is asked to repeat gradually increasing spans of numbers (e.g., 2-7-4), first forwards and then backwards. We combined backwards and forwards DS scores as done in previous work (47). The final DS score ranges from 0 to 30. We additionally evaluated a global cognitive composite score, which was calculated as an average of the z-scores of the DSC, CASI and DS tests.

© 2024 Domingo-Relloso A et al. *JAMA Network Open.*

eTable 1. Participant characteristics of included participants compared to the full sample.

Variables	Included (N=4,186)[a]	Full sample (N=6,766)
Age (years)	60 (52, 67)	62 (53, 70)
Sex (%)		
Female	52.6	52.8
Male	47.4	47.2
Education level (%)		
≤ High School	30.8	36.2
Some College	24.2	23.5
> College	45.0	40.3
BMI (kg/m^2)	27.5 (24.5, 31.1)	27.6 (24.6, 31.2)
eGFR ($mL/min/1.73m^2$)	78.7 (68.3, 89.4)	77.7 (66.8, 88.4)
Smoking (%)		
Never	51.2	50.3
Former	36.6	36.7
Current	12.2	13.0

[a] Participants with metals and cognitive outcomes data.

© 2024 Domingo-Relloso A et al. *JAMA Network Open.*

eTable 2. Median (25th, 75th percentiles) of cognitive tests measured in MESA participants in 2010-2012 by quartiles of urine metals per g creatinine measured in 2000-2002.

Metal quartiles	Digit Symbol Coding (N= 3,819)	Cognitive Abilities Screening Instrument (N=3,918)	Digit Span Test (N=4,276)
Arsenic µg/g			
< 0.21	50 (38, 62)	90.5 (84.4, 95.0)	15 (12, 18)
0.21 – 0.33	52 (39, 63)	90.4 (85.0, 94.5)	15 (12, 18)
0.33 – 0.53	51 (38, 65)	89.5 (84.0, 94.4)	15 (12, 18)
> 0.53	50 (37, 64)	88.5 (83.4, 94.4)	15 (12, 19)
Cadmium µg/g			
< 0.35	52 (40, 65)	91.5 (85.4, 95.5)	15 (12, 18)
0.35-0.51	52 (39, 65)	89.9 (84.3, 94.5)	15 (12, 18)
0.51-0.76	50 (37, 62)	89.5 (84.5, 94.5)	15 (12, 18)
> 0.76	49 (37, 61)	88.4 (82.9, 93.0)	15 (12, 18)
Cobalt µg/g			
< 0.28	50 (38, 63)	90 (84.4, 94.5)	15 (12, 18)
0.28 – 0.38	51 (40, 64)	90.3 (84.1, 94.9)	15 (12, 18)
0.38 – 0.55	51 (38, 63)	89.8 (84, 94.4)	15 (12, 18)
> 0.55	51 (36, 64)	89.4 (84, 94.5)	15 (12, 18)
Copper µg/g			
< 10	52 (40, 64)	91.0 (85.4, 95.0)	15 (12, 18)
10-12.2	53 (40, 64)	90.0 (84.2, 95.0)	15 (12, 18)
12.2-15.5	51 (37, 63)	89.9 (84.0, 94.4)	15 (12, 18)
> 15.5	49 (35, 62)	88.4 (83.0, 94.0)	15 (12, 18)
Lead µg/g			
< 0.66	52 (39, 65)	90.5 (84.5, 95)	15 (12, 18)
0.66-0.91	52 (38, 64)	89.5 (84.3, 94.4)	15 (12, 18)
0.91-1.31	51 (38, 63)	90.0 (84.7, 94.8)	15 (12, 18)
> 1.31	49 (36, 62)	88.9 (83.0, 94.0)	15 (12, 18)
Manganese µg/g			
< 0.16	52 (40, 64)	90.4 (84.5, 95.0)	15 (12, 18)
0.16 – 0.26	51 (38, 64)	89.9 (83.5, 94.5)	15 (12, 18)
0.26 – 0.44	51 (38, 63)	89.9 (84.0, 94.4)	15 (12, 18)
> 0.44	50 (37, 63)	89.0 (84.4, 94.5)	15 (12, 18)
Tungsten µg/g			
< 0.036	50 (37, 63)	89.9 (83.5, 94.5)	15 (12, 18)
0.036 – 0.059	50 (38, 63)	90.0 (84.5, 94.5)	15 (12, 18)
0.059 – 0.10	52 (39, 64)	89.6 (83.9, 94.5)	15 (12, 19)
> 0.10	51 (38, 64)	89.7 (84.4, 94.4)	15 (12, 18)
Uranium µg/g			
< 0.003	51 (38, 62)	90.5 (84.5, 95.0)	15 (12, 18)
0.003 – 0.005	51 (38, 64)	90.4 (84.5, 95.0)	15 (12, 18)
0.005 – 0.010	51 (38, 63)	89.9 (84.4, 94.5)	15 (12, 18)
> 0.010	50 (39, 65)	88.5 (83.0, 93.0)	15 (12, 19)
Zinc µg/g			
< 346	54 (42, 67)	91.5 (85.4, 95.5)	15 (13, 19)

© 2024 Domingo-Relloso A et al. *JAMA Network Open.*

346 – 518	52 (39, 64)	90 (84.5, 94.5)	15 (12, 18)
518 – 775	50 (37, 61)	89.4 (83.5, 94.0)	14 (12, 18)
> 775	47 (34, 60)	88.5 (83.0, 93.9)	14 (12, 18)

Abbreviations: Q1, first quartile; Q2, second quartile; Q3, third quartile; Q4, fourth quartile.

© 2024 Domingo-Relloso A et al. *JAMA Network Open.*

eTable 3. Mean difference (95% CI) of z-score cognitive tests measured in 2010-2012 per one interquartile range increase in log-transformed urine metal levels (μg/g creatinine) measured in 2000-2002.

	DSC (N=3,819)	CASI (N=3,918)	DS (N=4,176)	Global cognitive composite score[a] (N=3,592)
Arsenic	-0.034 (-0.068, 0.000)[b]	0.005 (-0.033, 0.043)	-0.005 (-0.043, 0.032)	-0.005 (-0.033, 0.022)
Cadmium	-0.021 (-0.060, 0.018)	0.041 (-0.003, 0.084)	0.002 (-0.040, 0.045)	0.001 (-0.030, 0.032)
Cobalt	-0.046 (-0.088, -0.004)[bc]	-0.016 (-0.050, 0.018)	-0.011 (-0.045, 0.023)	-0.034 (-0.068, 0.000)[bc]
Copper	-0.047 (-0.074, -0.019)[bc]	-0.030 (-0.062, 0.002)	-0.043 (-0.073, -0.012)[b]	-0.028 (-0.049, -0.006)[bc]
Lead	0.000 (-0.031, 0.032)	0.053 (0.005, 0.101)[bc]	0.007 (-0.028, 0.041)	0.035 (0.000, 0.069)[bc]
Manganese	-0.013 (-0.043, 0.017)	-0.006 (-0.039, 0.027)	0.006 (-0.027, 0.038)	-0.009 (-0.033, 0.015)
Tungsten	-0.019 (-0.049, 0.010)	0.012 (-0.021, 0.046)	-0.026 (-0.059, 0.007)	-0.010 (-0.034, 0.014)
Uranium	-0.039 (-0.076, -0.001)[b]	-0.003 (-0.044, 0.039)	-0.001 (-0.042, 0.039)	-0.009 (-0.039, 0.021)
Zinc	-0.034 (-0.063, -0.006)[b]	0.003 (-0.028, 0.035)	-0.020 (-0.051, 0.011)	-0.024 (-0.046, -0.001)[b]

Abbreviations: DSC, Digit Symbol Coding; CASI, Cognitive Abilities Screening Instrument; DS, Digit Span.
Model adjusted for age, sex, study site, education, estimated glomerular filtration rate, smoking status, body mass index, language of test administration, depression score and *APOE*4 allele carrier status.
The standard deviations were 18.6 for of DSC, 6.8 for the global cognitive score, 7.7 for CASI and 4.6 for DS.
P-values adjusted for multiple comparisons using the Benjamini-Hochberg approach for DSC were 0.08 for arsenic, 0.37 for cadmium, 0.08 for cobalt, 0.01 for copper, 0.99 for lead, 0.43 for manganese, 0.30 for tungsten, 0.08 for uranium and 0.08 for zinc. For the global cognitive score, 0.79 for arsenic, 0.95 for cadmium, 0.11 for cobalt, 0.11 for copper, 0.11 for lead, 0.68 for manganese, 0.68 for tungsten, 0.71 for uranium and 0.11 for zinc. For CASI, 0.90 for arsenic, 0.19 for cadmium, 0.81 for cobalt, 0.19 for copper, 0.19 for lead, 0.90 for manganese, 0.83 for tungsten, 0.90 for uranium and 0.90 for zinc. For DS, 0.94 for arsenic, 0.94 for cadmium, 0.94 for cobalt, 0.06 for copper, 0.94 for lead, 0.94 for manganese, 0.54 for tungsten, 0.94 for uranium and 0.62 for zinc.
[a] Global cognitive composite score is calculated as the average of the scores of the Digit Symbol Coding, the Cognitive Abilities Screening Instrument and the Digit Span tests.
[b] Metals that show statistically significant effects on DSC.
[c] Effect estimates and p-values obtained from a model using restricted quadratic splines with knots in the 10th, 50th, and 90th percentiles of the metal distribution.

© 2024 Domingo-Relloso A et al. *JAMA Network Open.*

eTable 4. Mean difference (95% CI) of z-score cognitive tests measured in 2010-2012 (exam 5) per one interquartile range increase in log-transformed urine non-priority metal levels measured in 2000-2002 (exam 1).

	DSC	CASI	DS	Global cognitive composite score[a]
Strontium	-0.002 (-0.036, 0.032)	-0.007 (-0.045, 0.031)	-0.004 (-0.041, 0.033)	0.001 (-0.026, 0.029)
Barium	0.017 (-0.015, 0.049)	0.023 (-0.013, 0.058)	0.013 (-0.022, 0.047)	0.011 (-0.014, 0.037)
Cesium	-0.009 (-0.047, 0.029)	0.059 (0.018, 0.1)[bc]	0.036 (-0.005, 0.077)	0.043 (0.013, 0.072)[bc]
Molybdenum	-0.024 (-0.054, 0.006)	-0.012 (-0.045, 0.021)	-0.026 (-0.058, 0.007)	-0.009 (-0.033, 0.015)
Thallium	-0.001 (-0.034, 0.033)	0.048 (0.002, 0.094)[bc]	0.016 (-0.02, 0.052)	0.007 (-0.019, 0.034)

Model adjusted for age, sex, study site, education, estimated glomerular filtration rate, smoking status, body mass index, language of test administration, depression score and *APOE*4 allele carrier status.

[a] Global cognitive composite score is calculated as the average of the scores of the Digit Symbol Coding, the Cognitive Abilities Screening Instrument and the Digit Span tests.

[b] Metals that show statistically significant effects on DSC.

[c] Effect estimates and p-values obtained from a model using restricted quadratic splines with knots in the 10th, 50th, and 90th percentiles of the metal distribution.

Abbreviations: DSC, Digit Symbol Coding; CASI, Cognitive Abilities Screening Instrument; DS, Digit Span test.

© 2024 Domingo-Relloso A et al. *JAMA Network Open.*

eTable 5. Mean difference (95% CI) of z-score Digit Symbol Coding measured in 2010-2012 per one interquartile range increase in log-transformed urine metal levels (μg/g creatinine) measured in 2000-2002 in MESA participants, overall and by *APOE4* carrier status, adjusted for cardiovascular disease risk factors.

	Overall (N=3,757)	No APOE-ε4 allele (N=2,722)	1-2 APOE-ε4 alleles (N=1,035)[a]	p-value for interaction[b]
Arsenic	-0.039 (-0.073, -0.005)[§]	-0.043 (-0.083, -0.003)[§]	-0.028 (-0.097, 0.040)	0.44
Cadmium	-0.017 (-0.056, 0.021)	-0.004 (-0.051, 0.043)	-0.043 (-0.111, 0.026)	0.20
Cobalt	-0.046 (-0.088, -0.004)*[§]	-0.048 (-0.084, -0.012)[§]	-0.046 (-0.106, 0.014)	0.67
Copper	-0.040 (-0.067, -0.013)*[§]	-0.046 (-0.079, -0.014)*[§]	-0.035 (-0.086, 0.016)	0.92
Lead	-0.006 (-0.038, 0.026)	0.013 (-0.024, 0.051)	-0.058 (-0.118, 0.001)	0.10
Manganese	-0.010 (-0.040, 0.019)	0.007 (-0.028, 0.042)	-0.052 (-0.109, 0.006)	0.08
Tungsten	-0.014 (-0.043, 0.016)	-0.012 (-0.046, 0.023)	-0.012 (-0.072, 0.047)	0.89
Uranium	-0.041 (-0.078, -0.004)[§]	-0.025 (-0.070, 0.020)	-0.079 (-0.145, -0.012)[§]	0.19
Zinc	-0.018 (-0.048, 0.011)	-0.002 (-0.036, 0.032)	-0.067 (-0.124, -0.009)[§]	0.04

Model adjusted for age, sex, race / ethnicity, study site, education, estimated glomerular filtration rate, smoking status, body mass index, language of test administration, depression score, LDL cholesterol, HDL cholesterol, lipid lowering medication, diabetes status, systolic blood pressure, and hypertension medication. The overall model is additionally adjusted for *APOE4* allele carrier status.
P-values adjusted for multiple comparisons using the Benjamini-Hochberg approach for the overall sample are 0.07 for arsenic, 0.49 for cadmium, 0.07 for cobalt, 0.03 for copper, 0.71 for lead, 0.55 for manganese, 0.49 for tungsten, 0.07 for uranium and 0.39 for zinc.

[a] Participants carrying either one or two *APOE4* alleles.

[b] P-value for interaction between carrying 1/2 *APOE*4 alleles and each metal.

[§] Metals that show statistically significant effects on DSC.

* Effect estimates and p-values obtained from a model using restricted quadratic splines with knots in the 10th, 50th, and 90th percentiles of the metal distribution.

© 2024 Domingo-Relloso A et al. *JAMA Network Open.*

eTable 6. Mean difference (95% CI) of z-score Digit Symbol Coding measured in 2010-2012 (exam 5) per one interquartile range increase in the mean between log-transformed urine metal levels measured in 2000-2002 (exam 1) and 2010-2012 (exam 5), as a measure of long-term exposure to metals.

	Overall (N=780)	No APOE-ε4 allele (N=569)	1-2 APOE-ε4 alleles (N=211)[a]	p-value for interaction[b]
Arsenic	-0.03 (-0.10, 0.05)	-0.05 (-0.14, 0.03)	0.04 (-0.10, 0.19)	0.42
Cadmium	-0.05 (-0.14, 0.04)	-0.03 (-0.14, 0.07)	-0.09 (-0.27, 0.09)	0.35
Cobalt	-0.06 (-0.12, 0.00)[§]	-0.10 (-0.20, 0.00)*[§]	-0.03 (-0.17, 0.11)	0.81
Copper	-0.06 (-0.11, 0.00)*[§]	-0.09 (-0.15, -0.02)*[§]	-0.02 (-0.16, 0.11)	0.70
Lead	0.11 (0.02, 0.20)*[§]	0.13 (0.03, 0.23)*[§]	-0.01 (-0.15, 0.12)	0.65
Manganese	-0.04 (-0.10, 0.02)	-0.02 (-0.10, 0.05)	-0.06 (-0.18, 0.06)	0.45
Tungsten	-0.07 (-0.13, 0.00)[§]	-0.09 (-0.17, -0.01)[§]	-0.03 (-0.15, 0.09)	0.75
Uranium	-0.07 (-0.16, 0.02)	-0.06 (-0.18, 0.05)	-0.130 (-0.3, 0.03)	0.21
Zinc	-0.10 (-0.17, -0.02)[§]	-0.10 (-0.18, -0.01)[§]	-0.11 (-0.26, 0.04)	0.63

Model adjusted for age, sex, study site, education, estimated glomerular filtration rate, smoking status, body mass index, language of test administration and depression score. The overall model is additionally adjusted for *APOE*4 allele carrier status.

P-values adjusted for multiple comparisons using the Benjamini-Hochberg approach for the overall sample are 0.47 for arsenic, 0.27 for cadmium, 0.11 for cobalt, 0.09 for copper, 0.06 for lead, 0.27 for manganese, 0.09 for tungsten, 0.20 for uranium and 0.06 for zinc.

[a] Participants carrying either one or two *APOE4* alleles.

[§] Metals that show statistically significant effects on DSC.

*For copper, lead, tungsten, and cobalt, because the dose-response was not linear, the effect estimates and p-values were obtained from a model using restricted quadratic splines with knots in the 10th, 50th, and 90th percentiles of the metal distribution.

© 2024 Domingo-Relloso A et al. *JAMA Network Open.*

eTable 7. Subdistribution hazards (95 %CIs) of possible *ICD*-based all cause dementia per one interquartile range increase in log-transformed urine metal levels calculated from the Fine-Gray model to evaluate competing risks for death.

	HR (95% CI)	p-value
Arsenic	1.21 (0.98, 1.49)[b]	0.08
Cadmium	1.09 (0.95, 1.25)	0.24
Cobalt	1.09 (0.93, 1.27)[b]	0.29
Copper	1.11 (1.01, 1.23)[ab]	0.03
Lead	0.94 (0.84, 1.04)	0.24
Manganese	1.02 (0.92, 1.14)	0.70
Tungsten	1.12 (1, 1.26)[a]	0.04
Uranium	1.32 (0.96, 1.8)[b]	0.08
Zinc	1.23 (1.04, 1.46)[ab]	0.02

Abbreviations: HR, hazard ratio.
Model adjusted for age, sex, race / ethnicity, study site, education, estimated glomerular filtration rate, smoking status and body mass index.
[a] Metals that show statistically significant effects on dementia
[b] Effect estimates and p-values obtained from a model using restricted quadratic splines with knots in the 10th, 50th, and 90th percentiles of the metal distribution.

© 2024 Domingo-Relloso A et al. *JAMA Network Open.*

eFigure 1. Flowchart of the Multi-Ethnic Study of Atherosclerosis participants.

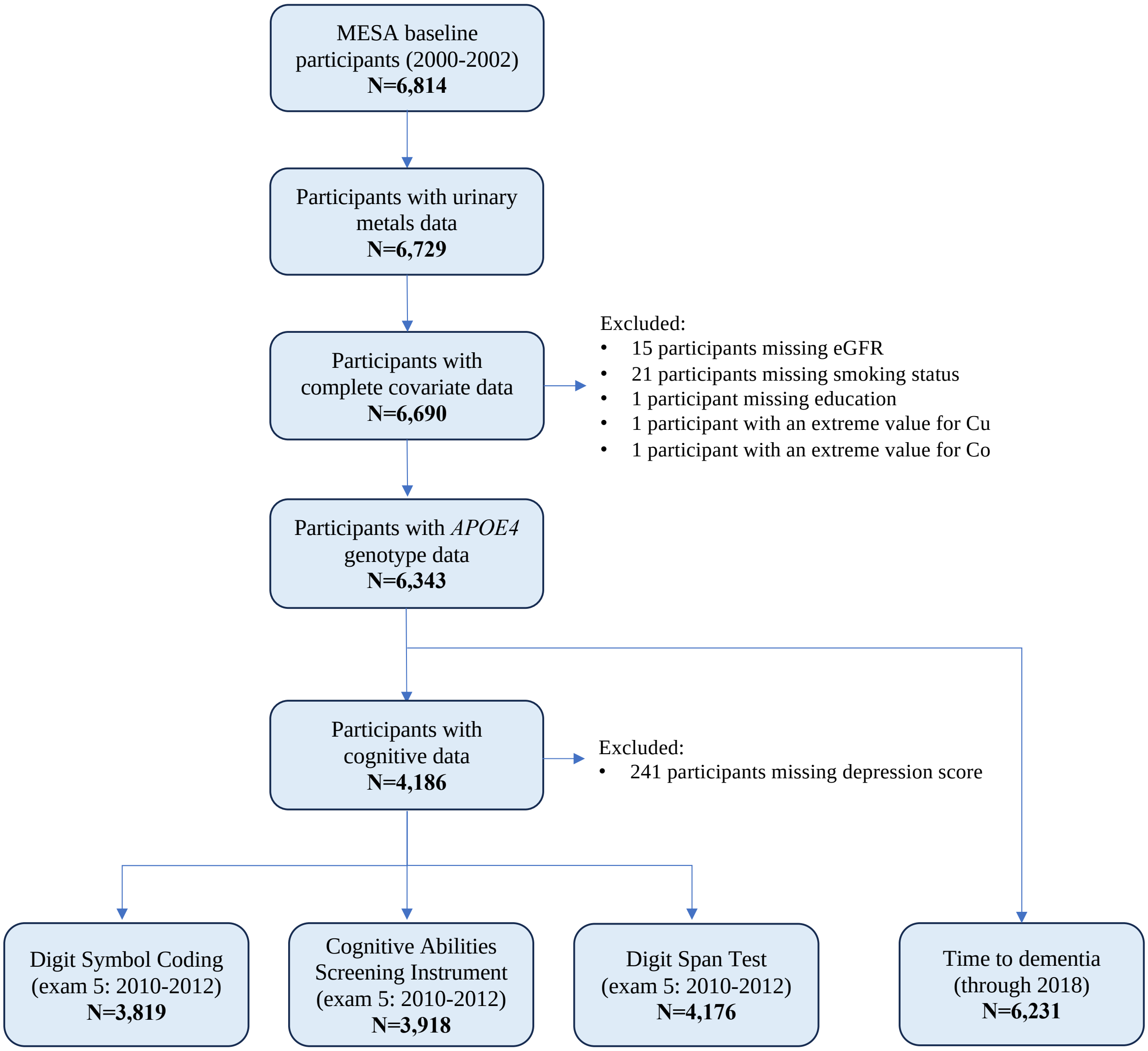

Abbreviations: MESA, Multi-Ethnic Study of Atherosclerosis; eGFR, estimated glomerular filtration rate; Cu, copper; Co, cobalt.

© 2024 Domingo-Relloso A et al. *JAMA Network Open.*

eFigure 2. Spearman correlations between metals.

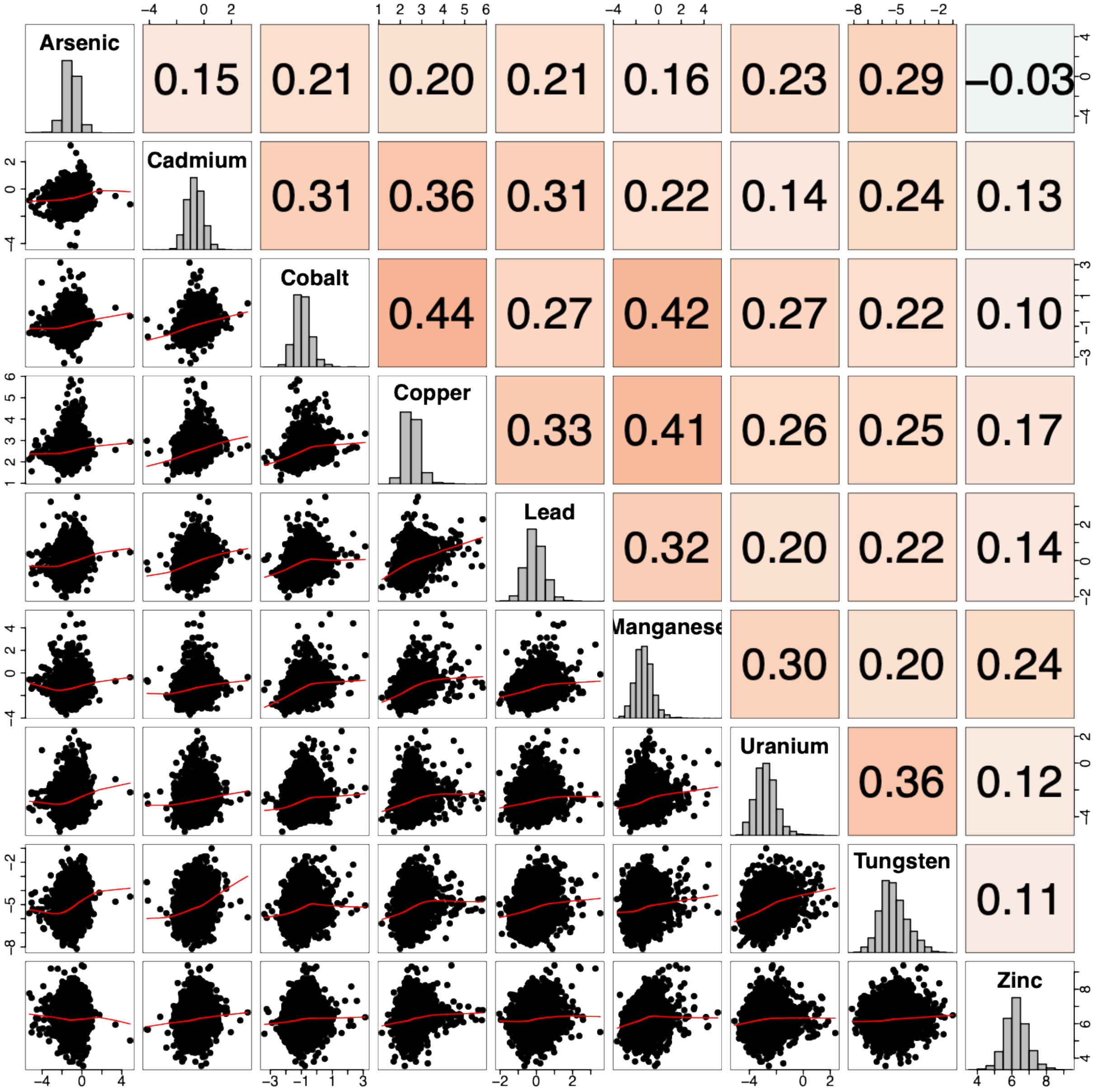

© 2024 Domingo-Relloso A et al. *JAMA Network Open.*

eFigure 3. Dose-response of the association between urinary metals and Digit Symbol Coding using restricted quadratic splines.

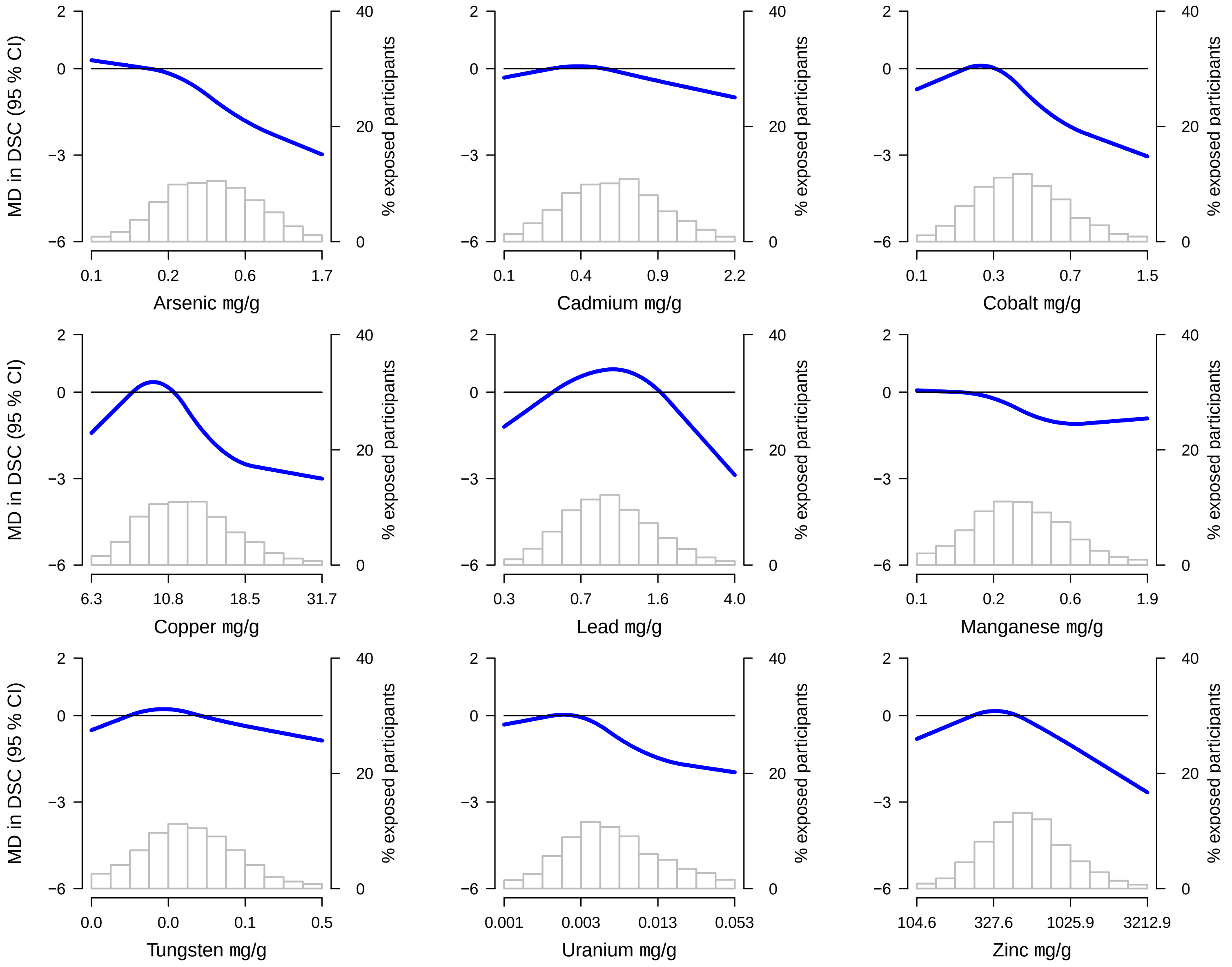

© 2024 Domingo-Relloso A et al. *JAMA Network Open.*

The plot is in semi-logarithmic scale. The values of the x axis are in logarithmic scale, but the axis labels are not log-transformed, and represent the real concentrations of urinary metals. The y axis is in z-score scale.
Abbreviations: MD, mean difference; CI, confidence interval.
The solid lines represent adjusted mean differences based on restricted quadratic splines for the log-transformed concentration of urinary metals, with knots set at 10th, 50th and 90th percentiles. The shadowed areas represent the upper and lower 95% confidence intervals. The reference was set at the 10th percentile. The x axis represents metal concentrations at their original scale. The y axis represents mean differences of the Digit Symbol Coding test, and the z axis represents the percentage of participants exposed to each range of metal concentrations in the displayed histogram.
Models were adjusted for age, sex, race / ethnicity, study site, education, estimated glomerular filtration rate, smoking status, body mass index, language of test administration, depression score and *APOE4* allele carrier status.

© 2024 Domingo-Relloso A et al. *JAMA Network Open.*

eFigure 4. Mean difference (95% CI) of z-score Digit Symbol Coding (DSC) for the individual urine metal distribution using Bayesian Kernel Machine Regression for A) Any *APOE4* allele carriers, B) No *APOE4* allele carriers.

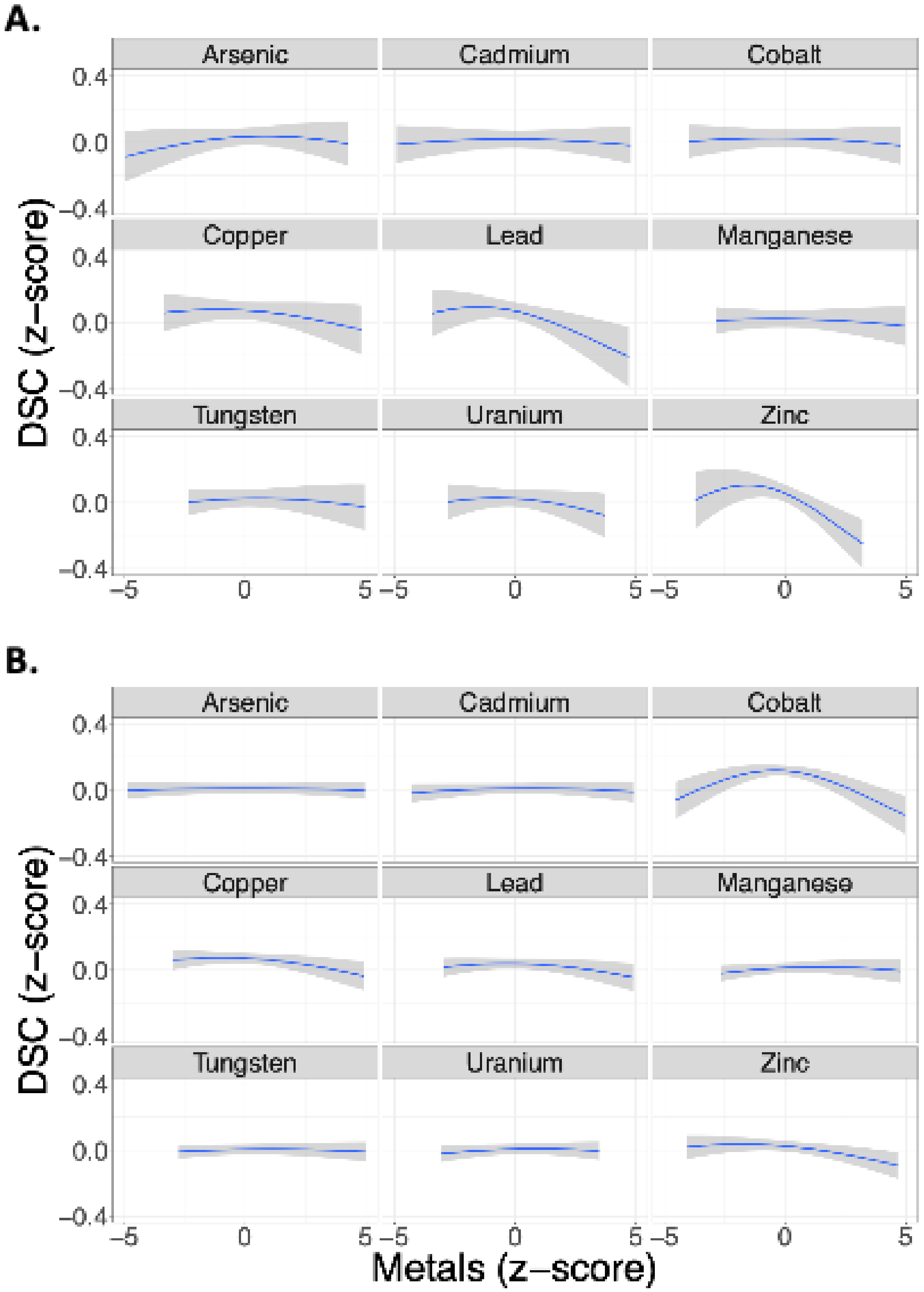

© 2024 Domingo-Relloso A et al. *JAMA Network Open.*

Posterior inclusion probabilities for participants carrying any *APOE*4 allele (N=1,058): arsenic 0.32, cadmium 0.19, cobalt 0.19, copper 0.35, lead 0.63, manganese 0.21, uranium 0.29, tungsten 0.28, and zinc 0.95. For participants carrying no *APOE*4 alleles (N=2,761): arsenic 0.09, cadmium 0.15, cobalt 0.79, copper 0.29, lead 0.33, manganese 0.25, uranium 0.23, tungsten 0.14, and zinc 0.42.

© 2024 Domingo-Relloso A et al. *JAMA Network Open.*
