# Supplementary material for: Urinary Metal Levels, Cognitive Test Performance, and Dementia in the Multi-Ethnic Study of Atherosclerosis
Source: JAMA Netw Open. 2024 Dec 2;7(12):e2448286. doi: 10.1001/jamanetworkopen.2024.48286 (PMC11612832; doi:10.1001/jamanetworkopen.2024.48286)
Supplement: Supplement 2. — Data Sharing Statement [file jamanetwopen-e2448286-s002.pdf]

Data Sharing Statement

Domingo-Relloso. Urinary Metal Levels, Cognitive Test Performance, and Dementia in the Multi-Ethnic Study of Atherosclerosis. *JAMA Netw Open*. Published December 02, 2024. doi:10.1001/jamanetworkopen.2024.48286

Data
Data available: No

Additional Information
Explanation for why data not available: The data used in this study cannot be publicly shared, however, it will be shared to investigators upon request through the Multi-Ethnic Study of Atherosclerosis website: https://www.mesa-nhlbi.org/. The complete code used in statistical analysis can be shared with investigators on request to the corresponding author.
